# Visual‐somatosensory integration as a novel behavioral marker of amyloid pathology

**DOI:** 10.1002/alz.14561

**Published:** 2025-03-07

**Authors:** Jeannette R. Mahoney, Emmeline Ayers, Joe Verghese

**Affiliations:** ^1^ Department of Neurology Division of Cognitive and Sensorimotor Aging Renaissance School of Medicine Stony Brook University Stony Brook New York USA

**Keywords:** amyloid pathology, APOE, behavioral markers, multisensory integration, sensorimotor processing

## Abstract

**INTRODUCTION:**

The ability to integrate information across sensory modalities is a vital aspect of everyday functioning and is linked to cognition. Increasing evidence suggests that Alzheimer's disease (AD) pathology manifests in sensory association areas before appearing in higher‐order cognitive areas. We examined the role of visual‐somatosensory integration (VSI) as a novel behavioral marker of AD‐associated amyloid pathology.

**METHODS:**

This cross‐sectional study included 243 adults (77 ± 6.5 years; 52% female) who completed the VSI test and AD biomarker assays. The *magnitude of VSI* was the *independent variable* and amyloid‐beta probability scores (APS; PrecivityAD^TM^) were the *dependent variable*. Cognitive status (normal, mild cognitive impairment, or AD) was assigned during case conferences.

**RESULTS:**

Linear regression revealed an inverse association between the *magnitude of VSI* and APS (*β* = −0.16; *p* ≤ 0.01). As cognitive impairment increased from normal to dementia, the *magnitude of VSI* decreased (*p* < 0.05).

**DISCUSSION:**

Findings provide support for VSI impairment as a new behavioral marker of AD‐associated amyloid pathology.

**Highlights:**

- Here we provide support for the *magnitude of VSI* as a novel behavioral marker of AD‐associated amyloid pathology given its significant association with an established, accurate, and reliable biomarker of AD pathology.
- Adults with normal cognition maintained the highest *magnitude of VSI* and brain amyloid negative scores.
- As cognitive impairment increased, the mean *magnitude of VSI* significantly decreased while amyloid probability scores (APS) increased. In fact, individuals with dementia revealed the lowest *magnitude of VSI* and the highest APS.
- Our research continues to emphasize the importance of successful multisensory integration in aging, where the establishment of future novel multisensory‐based interventions aimed at preventing disability and optimizing independence could prove valuable.

## 1 BACKGROUND

Alzheimer's disease (AD), the most common cause of dementia, affects ∼6.7 million older Americans.^1^ Sensory impairments have been associated with cognitive decline and increased dementia risk.^2^, ^3^ In fact, the National Institute on Aging (NIA) has recognized that functional changes in both sensory and motor systems modulate the progression of AD, but research is still in its infancy.^4^

The ability to integrate information across sensory modalities is a vital aspect of everyday functioning. Multisensory integration (MSI) refers to a process by which information across sensory systems is combined and integrated in the brain. The phenomenon occurs when simultaneously presented sensory information (e.g., visual and somatosensory) yields faster responses than responses to either constituent condition presented alone. Researchers argue that our brains are designed to simultaneously process information from multiple sensory inputs to produce the most appropriate response to environmental cues.^5^ Studies in primates and young adults reveal that MSI behavior is regulated by areas in the prefrontal cortex (PFC), including dorsomedial and ventrolateral subregions.^6^, ^7^

While the existence of limited age‐related MSI investigations and lack of clinical‐translational findings have been highlighted as major knowledge gaps,^8^, ^9^, ^10^, ^11^ significant efforts are underway to bridge these “gaps.” Age‐related changes in MSI, including auditory‐visual integration processes, have been associated with delayed time windows of MSI^12^ and greater susceptibility to sound‐induced flash illusion.^13^, ^14^, ^15^, ^16^, ^17^ Such changes in MSI have also been associated with worse postural stability, falls, & decreased physical activity^13^, ^14^, ^15^, ^16^, ^17^ and cognitive impairments.^18^ Results from our simple reaction time (RT) test reveals that poor visual‐somatosensory integration (VSI) is linked to worse balance,^19^, ^20^ slower gait,^21^ and increased falls.^20^

Compared to healthy adults, individuals with cognitive impairments demonstrate different psychophysical/behavioral profiles which can encompass longer response times, wider temporal‐binding windows, impairments in attention, and display differential multisensory enhancements.^12^, ^18^, ^22^, ^23^, ^24^ Our work reveals that worse *magnitude of VSI* is associated with slower gait and that this association is stronger in those with cognitive decline compared to those with normal cognition.^24^ More specifically, poor VSI is associated with poor attention‐based performance,^24^, ^25^ which jointly rely on PFC regions known to be compromised in AD. However, direct links between MSI and AD pathology are currently lacking. Thus, further examination is warranted to parse declines associated with “normal” aging, from impairments associated with declines in MSI and neurodegenerative diseases.

There are many  risk factors indicative of AD pathology, however, the greatest determinants include: increased age, AD‐associated genetics, and family history of AD.^26^ In general, AD is associated with a build‐up of proteins in the brain, namely amyloid‐beta (Aβ) in the form of plaques^27^ outside the neurons and tau (T) in the form of neurofibrillary tangles inside the neurons. In terms of genetics, the apolipoprotein E (APOE) ε4 allele is the most prevalent genetic risk factor for AD and is associated with increased brain Aβ deposition in the early^28^ and late^29^ stages of cognitive decline. According to the NIA, these proteins often lead to AD‐associated neurodegeneration like loss of synaptic connections that interfere with neuronal communication, often causing AD hallmarks of cognitive decline.^30^ Studies have demonstrated associations between Aβ and sensory impairments, but to our knowledge, none have explicitly examined its association with VSI performance.

Evidence reveals that AD‐associated amyloid pathology accumulates in sensory‐association areas of the brain well before higher‐order cognitive areas like PFC.^27^ As well, research reveals that older adults with increased amyloid deposition do not necessarily meet the criteria for cognitive impairment.^31^ However, Zhang and colleagues postulate that “*combined sensory impairment and the progression of AD may establish a cyclical relationship that mutually perpetuates each condition.”* ^32^ Here, we contend that the ability to integrate multisensory information offers promising potential as a behavioral marker given its associations with both cognitive and motor outcomes in older adults with and without cognitive impairments. The main objective of the current study is to establish whether VSI is a novel behavioral marker of AD‐associated amyloid pathology by assessing its association with accurate, reliable, and robust risk factors for AD pathology captured by Aβ probability scores (APS; PrecivityAD^TM^ ^33^).

## 2 METHODS

### 2.1 Participants

Two‐hundred‐forty‐three participants (mean age 76.87 ± 6.50 years; 52% female) enrolled in the longitudinal Central Control of Mobility in Aging (CCMA; R01AG036921) study who completed all of the following study procedures were included in the current study: (1) a multi‐disciplinary case conference where all clinical and cognitive data were reviewed to determine cognitive status in consensus; (2) a multisensory simple RT test (i.e., the VSI experiment described below); and who (3) provided a blood specimen as part of the study requirements. Each participant was only represented once (i.e., the earliest study wave with complete and valid data available for all abovementioned study procedures). Eligibility criteria for the parent CCMA study, which ran between June 2011 and June 2018, required that participants be 65 years of age and older, reside in lower Westchester County, and speak English. Exclusion criteria included inability to independently ambulate, dementia diagnosis at baseline (assessed using reliable and valid cut‐score of ≥ 2 on the AD8 Dementia Screening Interview cutoff score^34^ and < 5 on the memory impairment screen [MIS]^35^), significant bilateral vision and/or hearing loss, active neurological or psychiatric disorders that would interfere with evaluations, recent or anticipated medical procedures that would affect mobility, and/or receiving hemodialysis treatment.

Participants were enrolled in yearly clinical, psychological, neuropsychological, sensory, and motor evaluations that typically took place over two testing sessions during a 2‐week study period, depending upon their availability. As part of the CCMA study, all participants were required to have bilateral visual acuity that was better or equal to 20/100 as measured by the Snellen eye chart. To be consistent with established practices in visual acuity assessment literature,^36^, ^37^ raw Snellen scores were converted into logarithm of minimal angle of resolution (logMAR) scores. Lower logMAR scores are indicative of better visual acuity (e.g., 20/20 = 0 logMAR while 20/80 = 0.6 logMAR). Additionally, this conversion allows for the use of visual acuity as a continuous variable.^36^, ^37^ According to accepted procedures, the best (e.g., highest) acuity screening results, regardless of eye (e.g., left or right), were used in all analyses.^36^, ^37^, ^38^, ^39^ This approach has been successfully applied in some of our previous work.^40^ Individuals unable to hear a 2000 Hz tone at 25 dB in both ears were not included in the current study.

Somatosensory acuity scores (i.e., vibratory thresholds) were unfortunately not available for all participants in the parent study. However, the presence or absence of peripheral neuropathy was assigned by study clinicians based on neurological examination and observation of walking patterns. Participants unable to feel the somatosensory stimulation from the VSI test and who were very likely to have peripheral neuropathy (even if the clinician did not endorse neuropathy) were not included in the current study. A unique feature of this multisensory experiment is that performance on the unisensory conditions (visual alone and somatosensory alone) is considered when calculating the *magnitude of VSI* (see *Calculating the Magnitude of VSI* section below). As such, one could argue that the unisensory performance on this specific test almost serves as its own control for the multisensory phenomenon, beyond what can be additionally accounted for in statistical analyses.

Global Health Status (GHS) scores (range 0–9) were obtained from dichotomous ratings (presence/absence) of physician‐diagnosed diabetes, chronic heart failure, arthritis, hypertension, depression, stroke, chronic obstructive pulmonary disease, angina, and myocardial infarction

**RESEARCH IN CONTEXT**

1. **Systematic review**: The ability to successfully integrate information across multiple sensory modalities is a vital aspect of everyday functioning that is often not formally examined in healthy and pathological aging.
2. **Interpretation**: Our research reveals *magnitude of visual‐somatosensory integration* (VSI) as a promising new, non‐cognitive, non‐invasive behavioral marker of Alzheimer's disease (AD) associated amyloid pathology.
3. **Future directions**: Our research continues to emphasize the importance of successful multisensory integration in aging, where the establishment of future novel multisensory‐based interventions aimed at preventing disability and optimizing independence could prove valuable.

### 2.2 Clinical evaluation

As part of the CCMA study, individuals participated in a yearly neuropsychological battery that provided a comprehensive assessment of cognitive function, which has been validated in our previous longitudinal studies.^41^ A multidisciplinary clinical team conducted yearly case conferences for each participant where all available demographic, neuropsychological, neurological, psychosocial, and functional test data were formally reviewed, and cognitive status (normal, mild cognitive impairment [MCI], or dementia) was assigned using established criteria. In the current sample of 243 older adults, the diagnoses were as follows: 205 normal cognition, 27 MCI, and 11 dementia. While the risk for AD was defined based on biological markers (see below), the association of VSI with cognitive status was also assessed to further highlight the clinical significance and impact of any finding.

### 2.3 Plasma‐based AD Biomarkers

As previously mentioned, the risk for AD is multifactorial, but the greatest risk factors are increased age, AD‐associated genetics, and family history of AD.^26^ Apolipoprotein E (APOE), namely the APOE‐ε4 allele, is the strongest and most prevalent genetic risk factor of AD, and is known to increase Aβ deposition in the brain, leading to neural disruptions associated with AD.^42^ Aβ is a pathological hallmark of AD, that is, known to increase with age^43^ and accumulates in the sensory association areas well before higher‐order cognitive areas like the PFC.^27^ PrecivityAD^TM^ Aβ probability scores incorporate these three critical AD risk factors: chronological age, plasma Aβ42:40 ratio, and plasma APOE proteotypes (identified using validated liquid chromatography‐tandem mass spectrometry analyses at C_2_N Diagnostics’ CAP‐CLIA‐ISO13485 certified and accredited laboratory [St. Louis, MO]) to concurrently identify the likelihood of brain amyloid plaques (probability scores ranging from 0 to 100).^44^ The analytical and clinical performance of the PrecivityAD^TM^ plasma test and APS cut scores have been documented,^33^, ^44^, ^45^ where APS scores less than 35 represent a low likelihood for the presence of brain amyloid (amyloid negative status; APS−); scores between 35 and 58 represent an intermediate (APS‐interm) status associated with a 50% likelihood for the presence of brain amyloid; and scores greater than 58 represents a high likelihood for the presence of brain amyloid (amyloid positive; APS+). The main objective of this study is to determine the association of amyloid markers with VSI performance. In order have ensure that the relationship between *magnitude VSI* and amyloid probability scores was indeed linked to amyloid pathology, rather than just “normal” aging, a sensitivity analysis was also conducted (see *Statistical Analysis* section below).

### 2.4 The VSI experiment

**FIGURE 1:**
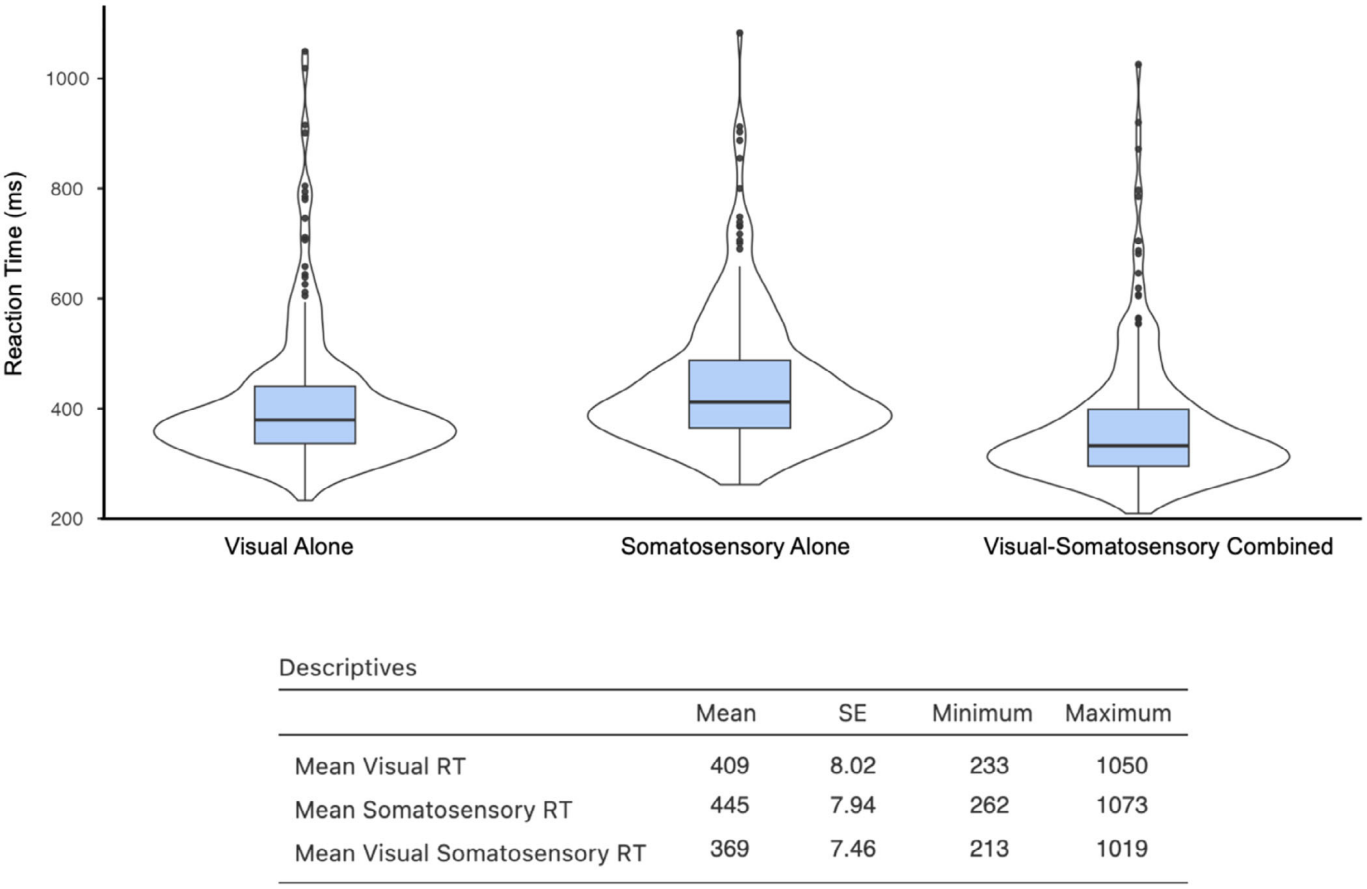
Descriptive table and violin plots of reaction time (RT) data to visual, somatosensory, and multisensory visual‐somatosensory stimuli

All participants completed our established VSI test.^46^ They were instructed to respond to unisensory (visual and somatosensory) and multisensory (simultaneous visual‐somatosensory) stimuli as quickly as possible by pressing a stationary foot pedal. The three stimulus conditions were presented randomly with equal frequency (15 trials of each stimulus condition per block). The experiment had a total of three blocks, each consisting of 45 trials yielding 135 trials in total (45 trials per stimulus condition). Anticipatory effects were prevented through the use of an inter‐stimulus‐interval that varied randomly from 1 to 3 s. Each block was separated by a 20 s break to reduce fatigue and facilitate concentration (see Ref.^47^ for details). Descriptive data and violin plots for RTs to all three sensory conditions are presented in Figure 1.

Performance accuracy was defined as the number of accurate stimulus detections divided by 45 trials per condition. As in our previous work, data trimming procedures were purposefully avoided so as to not bias the distribution of RT data, and RTs for all inaccurate (i.e., omitted) trials were set to infinity.^46^ Given the inclusion of adults with MCI and dementia, the average performance level to be included in the current study was set at 60%: note that, on average participants were 88% accurate or better at responding to all sensory conditions, irrespective of their cognitive status.

Visual and somatosensory stimuli were delivered alone and simultaneously through a custom‐built stimulus generator (Zenometrics, LLC; Peekskill, NY, USA) that consisted of two control boxes (one for each hand), each housing a 15.88 cm diameter blue light emitting diodes (LEDs; 325 LUX) and a 30.48 mm × 20.32 mm × 12.70 mm plastic housing containing a 12 mm vibrator motor with 0.8G vibration amplitude oscillating at 110 Hz. A TTL (transistor‐transistor‐logic, 5 V, duration 100 ms) pulse was used to trigger all sensory stimuli through E‐Prime 2.0 software. Control boxes were mounted to an experimental apparatus, which participants rested their hands upon comfortably, with index fingers placed over the vibratory motors on the back of the box and their thumb on the front of the box. A third dummy control box was placed in the center of the actual control boxes, at an equidistant length (28 cm), and contained a bull's eye sticker with a central circle of 0.4 cm diameter that served as the fixation point. To ensure that the somatosensory stimuli were inaudible, each participant was provided with headphones over which continuous white noise was played.

### 2.5 Calculating the *magnitude of VSI*

The methods employed for quantifying *magnitude of VSI* have been outlined extensively in our tutorial.^46^ Briefly, robust probability (P) models that compare the cumulative distribution function (CDF) of combined unisensory visual (V) and unisensory somatosensory (S) RTs with an upper limit of one (min [P(RT_V_ ≤ *t*) + P(RT_S_ ≤ *t*)], 1) to the CDF of multisensory VS RTs [P(RT_VS_ ≤ *t*)] were used.^48^, ^49^ For any latency *t*, the race model inequality (RMI) *holds* when the CDF of the **actual** multisensory condition [P(RT_VS_ ≤ *t*)] is less than or equal to the **predicted CDF** (min [P(RT_V_ ≤ *t*) + P(RT_S_ ≤ *t*)], 1). When the **actual CDF** is greater than the **predicted CDF**, the RMI is *rejected*, and the RT facilitation is the result of multisensory interactions that allow signals from redundant information to integrate or combine non‐linearly. Differences between **actual** and **predicted** CDFs are plotted to examine MSI effects (see also Ref.^46^).

**FIGURE 2:**
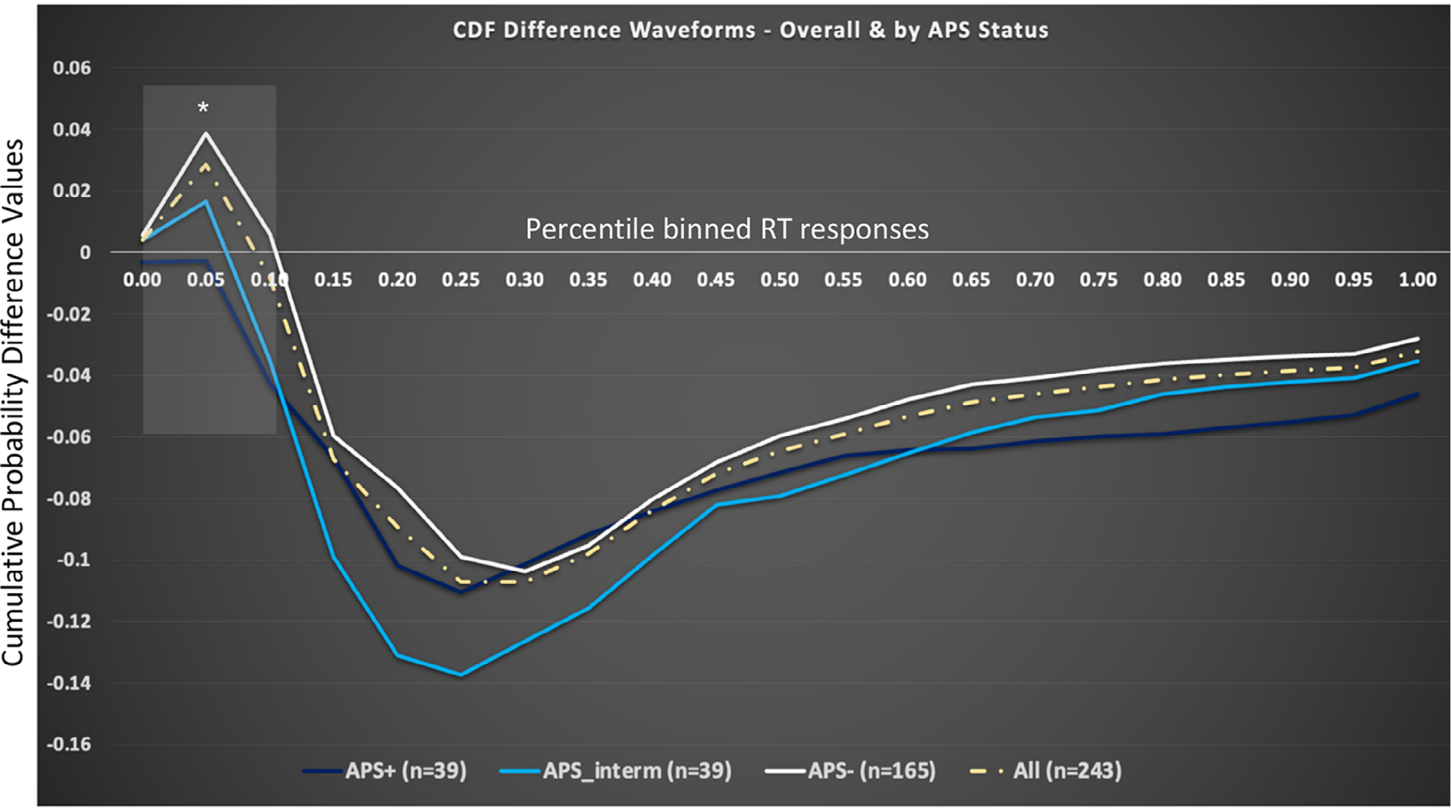
Cumulative distribution function (CDF) difference waveforms (indicative of VSI) for overall study group (yellow dashed trace) and by APS group (**Negative** [white trace]; **Intermediate** [cyan trace], and **Positive** [navy trace])

As in our most recent work,^20^, ^21^, ^24^ the *area under the curve* (AUC) during the violated percentile bins (i.e., bins that have positive values indicative of MSI for the study sample) served as a continuous measure of the *magnitude of VSI*. Figure 2 depicts CDF difference waveforms for the overall group (yellow dashed trace), where the x‐axis represents the percentile binned RT responses, and the y‐axis represents the cumulative probability difference values. Figure 2 reveals a clear violation of the RMI (positive values for bins 0.00–0.01). Thus, the fastest 10^th^ % of RTs represent the violated percentile bins for this cohort (highlighted in the gray box). For reference, individuals with positive cumulative probability difference wave values during the violated percentile bins (used to calculate AUC) are considered “*integrators*,” while those with negative difference wave values during the violated percentile bins are considered “*non‐integrators*.” Figure 2 also depicts the CDF difference waveforms for each of the three APS groups (APS− [white trace]; APS‐intermediate [cyan trace]; and APS+ [navy trace] groups).

### 2.6 Statistical analyses

Data were inspected descriptively and graphically, and the normality of model assumptions was formally tested for the independent and dependent measures using Shapiro–Wilk tests. Results revealed that the distribution of the *magnitude of VSI* did not show evidence of non‐normality (*W *= 0.992, *p*‐value = 0.18). However, the distribution of APS scores was in fact not normally distributed (*W *= 0.852, *p*‐value < 0.01), and therefore a natural log transformation was applied. Given APS scores of zero are possible and were present in the current dataset, a constant value of 1 was added to all scores before the log transformation. All statistical analyses performed utilized the *natural log of the APS +1* variable.

Descriptive statistics were calculated for continuous (M ± SD) and nominal variables (%) and are presented in Table 1 for the overall group (*n* = 243) and parsed by the APS group. All analyses were run using IBM's Statistical Package for the Social Sciences (SPSS) Version 29.

**TABLE 1:** Demographic and clinical characteristics overall and by APS status group^a^

Variable	All (*n* = 243)	APS− (*n* = 165)	APS_interm (*n* = 39)	APS+ (*n* = 39)	^b^ *p*‐ value
Age (years)	77 ± 6.50 (65–93)	75 ± 5.40 (65–93)	78 ± 6.43 (65–91)	84 ± 6.06 (72–92)	0.00
Gender (% female)	52	56	49	41	0.23
Ethnicity (% White)	78	72	87	95	0.00
Education (years)	14.97 ± 2.99 (2–21)	15.11 ± 2.86 (8–21)	15.26 ± 2.77 (12–21)	14.08 ± 3.58 (2–21)	0.12
GHS (0–9)	1.10 ± 0.98 (0–4)	1.08 ± 0.97 (0–4)	1.28 ± 1.03 (0–3)	1.00 ± 0.97 (0–4)	0.40
Visual acuity	0.26 ± 0.1 (−0.13–0.70)	0.24 ± 0.15 (−0.13–0.70)	0.29 ± 0.16 (0.00–0.54)	0.29 ± 0.12 (0.00–0.54)	0.10
% with neuropathy	6	3	10	13	0.03
Overall reaction time (ms)	407 ± 116 (243–945)	397 ± 107 (248–945)	445 ± 146 (243–944)	412 ± 116 (307–897)	0.07
Magnitude of VSI	0.03 ± 0.13 (−0.36–0.41)	0.04 ± 0.13 (−0.31–0.41)	0.00 ± 0.13 (−0.36–0.27)	−0.03 ± 0.14 (−0.32–0.29)	0.00
Plasma Aß42:40 ratio	.099 ± .010 (0.068–0.138)	.104 ± .008 (0.089–0.138)	.092 ± .004 (0.085–0.102)	.085 ± .006 (0.068–0.103)	0.00
Amyloid‐ß probability score	28.04 ± 27.94 (0–97)	11.27 ± 9.57 (0–34)	46.28 ± 6.20 (35–57)	80.77 ± 10.93 (59–97)	0.00
% APOE ε4 carriers	15	9	23	33	0.00
% Cognitively normal	84	88	87	67	0.01

Abbreviations: APOE, apolipoprotein E; GHS, Global Health Status; VSI, visual‐somatosensory integration.

^a^ Values are presented as mean ± SD for continuous and % for nominal variables.

^b^ *p*‐values for between group (APS status) one‐way ANOVA or chi‐square analyses.

A linear regression analysis assessed the association of *magnitude of VSI* (independent variable) with natural log transformed APS (dependent variable). The regression was run unadjusted first, and then adjusted for key covariates associated with AD pathology including: gender (female vs. male); ethnicity (White individuals vs. rest); years of education; GHS score; visual acuity (LogMAR values); presence of peripheral neuropathy; overall RT on the VSI test; and consensus case diagnosis (dementia, MCI or normal). Note that chronological age, Aß42:40 ratio, and presence of APOE‐ε4 were purposefully excluded as covariates in our regression model as they are inherent in the derivation of the APS.

To address concerns about whether the relationship between the *magnitude of VSI* (independent variable) and amyloid pathology could be due to “normal” aging, we ran an additional sensitivity analysis using the Aβ42:40 ratio. This analysis considered the normality of the distribution of the Aβ42:40 ratio (where five extreme outliers were excluded) and assessed the association of the *magnitude of VSI* (independent variable) with the Aβ42:40 ratio (dependent variable). AD pathology‐associated covariates entered in this adjusted model included age, gender (female vs. male); ethnicity (White individuals vs. rest); years of education; GHS score; visual acuity (LogMAR values); presence of peripheral neuropathy; overall RT on the VSI test; and consensus case diagnosis (dementia, MCI or normal).

## 3 RESULTS

Demographic information is presented in Table 1 for the overall cohort and by APS groups (APS−, APS‐intermediate, and APS+ categories). There were significant APS group differences in age, percentage of White individuals, percentage of individuals with neuropathy, *magnitude of VSI*, Aß42:40 ratio, percentage of APOEε4 carriers, and cognitive status by the APS group. Individuals in the APS+ group were older, had more neuropathy, less *magnitude of VSI*, lower Aß42:40 concentration levels, and a greater likelihood of being a White individual, cognitively impaired, and an APOEε4 carrier compared to the APS− and APS‐intermediate groups.

Linear regression results (Table 2) reveal strong inverse associations between the *magnitude of VSI* and APS (*β* = −0.16; *p* ≤ 0.01), even after adjusting for critical covariates including: gender, ethnicity, education level, medical comorbidities, visual acuity, presence peripheral neuropathy, overall RT on the VSI experiment, and cognitive diagnoses. Results indicate that older adults with increased AD‐associated amyloid pathology (i.e., higher APS scores) manifest significantly worse *magnitude of VSI*. Compared to APS− individuals, combined individuals in the APS‐intermediate and APS+ brain amyloid groups were 94% (OR = 1.942) more likely to demonstrate no (zero) or negative *magnitude of VSI* values (95% CI: 1.12–3.36; *χ* ^2^ = 0.02). A fully adjusted sensitivity analysis (*n* = 238) revealed significant associations between the *magnitude of VSI* and Aβ42:40 concentration levels (*β* = 0.13, *p* = 0.03; see Table 3).

**TABLE 2:** Unadjusted and adjusted linear regression results: association of magnitude of VSI with natural log‐transformed APS (*n* = 243)

		Unstandardized coefficients		Standardized coefficients			95.0% confidence interval for *ß*	
Model^a^	Parameter	*ß*	Std. error	Beta	*t*	Sig.	Lower bound	Upper bound
1	Magnitude of VSI	−1.45	0.61	−0.15	−2.36	0.02	−2.65	−0.24
2	Magnitude of VSI	−1.50	0.59	−0.16	−2.55	0.01	−2.65	−0.34
	Gender	−0.48	0.16	−0.19	−3.03	0.00	−0.80	−0.17
	Ethnicity	−0.93	0.19	−0.31	−4.99	<0.001	−1.30	−0.57
	Years of education	−0.03	0.03	−0.06	−0.95	0.34	−0.08	0.03
	GHS	0.06	0.08	0.05	0.81	0.42	−0.09	0.22
	Visual acuity	0.64	0.53	0.08	1.21	0.23	−0.40	1.68
	Neuropathy	0.43	0.34	0.08	1.28	0.20	−0.23	1.10
	Overall reaction time	0.00	0.00	0.02	0.24	0.81	0.00	0.00
	Consensus diagnosis	0.03	0.05	0.05	0.70	0.48	−0.06	0.12

Abbreviation: GHS, Global Health Status; VSI, visual‐somatosensory integration.

^a^ Dependent variable: Natural log (Aß probability score + 1).

**TABLE 3:** Fully‐adjusted sensitivity analysis: association of magnitude of VSI with Aβ42:40 concentration levels (*n* = 238)

		Unstandardized coefficients		Standardized coefficients			95.0% Confidence interval for *ß*	
Model^a^	Parameter	*ß*	Std. error	Beta	*t*	Sig.	Lower bound	Upper bound
1	Magnitude of VSI	0.01	0.00	0.13	2.22	0.03	0.00	0.02
	Age	0.00	0.00	−0.23	−3.66	<0.001	0.00	0.00
	Gender	0.01	0.00	0.25	4.00	<0.001	0.00	0.01
	Ethnicity	0.01	0.00	0.26	4.27	<0.001	0.00	0.01
	Years of education	0.00	0.00	0.03	0.56	0.58	0.00	0.00
	GHS	0.00	0.00	−0.02	−0.35	0.73	0.00	0.00
	Visual acuity	0.00	0.00	−0.05	−0.86	0.39	−0.01	0.00
	Neuropathy	0.00	0.00	−0.11	−1.75	0.08	−0.01	0.00
	Overall reaction time	0.00	0.00	0.02	0.23	0.82	0.00	0.00
	Consensus diagnosis	0.00	0.00	0.03	0.41	0.68	0.00	0.00

Abbreviations: GHS, Global Health Status; VSI, visual‐somatosensory integration.

^a^ Dependent variable: Aβ42:40 concentration levels.

**FIGURE 3:**
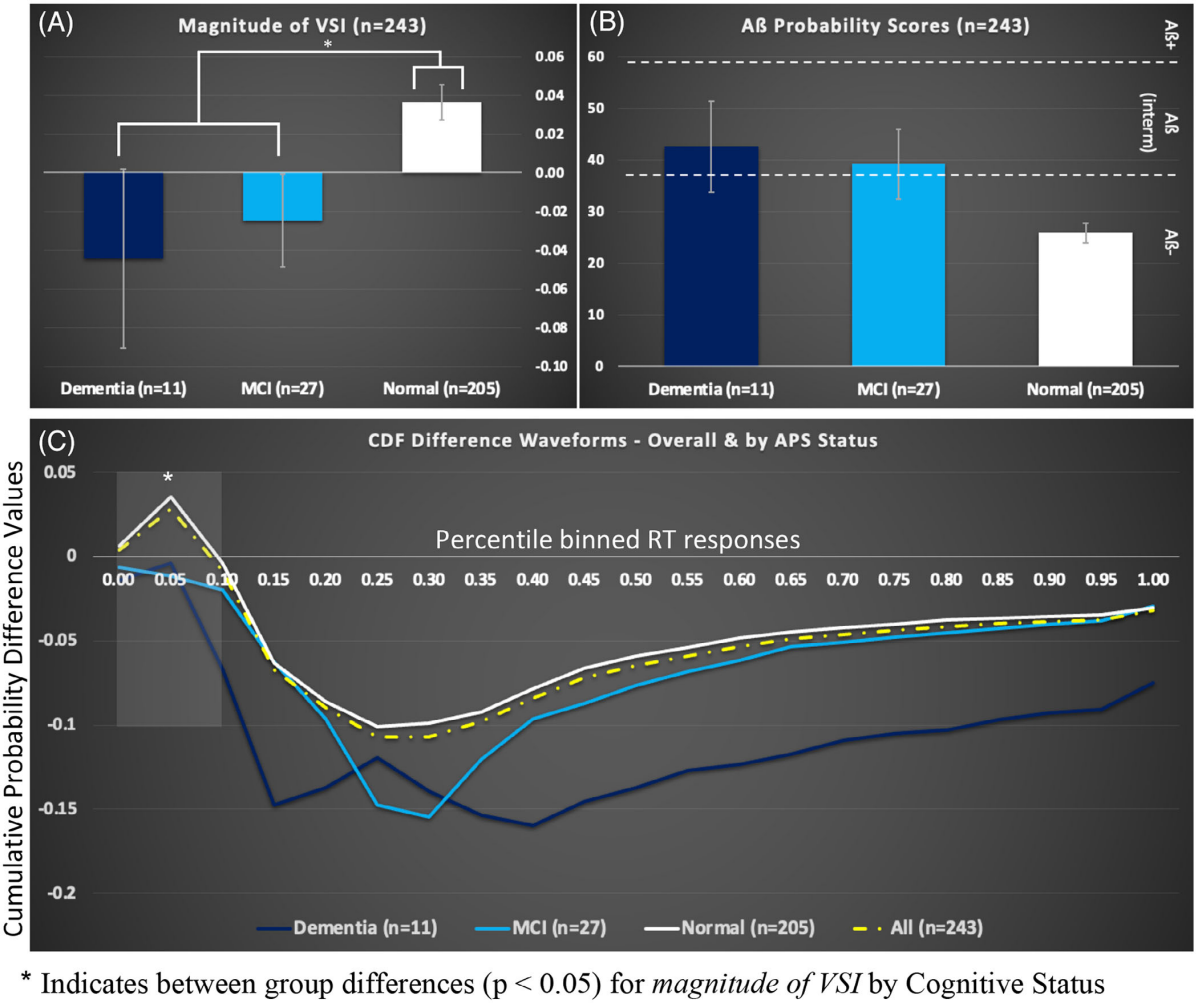
Color‐coded graphs indicating (A) Mean *magnitude of VSI*; (B) mean AB Probability Score; and (C) CDF Difference Waveforms ‐ Overall & by Cognitive Diagnosis (**Normal** [white]; **MCI** [cyan], and **Dementia** [navy]). Error bars represent the standard error of the mean. Overall study group (yellow dashed trace) was purposefully repeated for reference

Figure 3 depicts mean *magnitude of VSI* (a) and APS (b) by case conferenced cognitive diagnoses. Error bars represent standard error of the mean values. Figure 3C depicts CDF difference waveforms for the overall group (yellow dashed trace) and for each of the three cognitive status groups (dementia [navy trace]; MCI [cyan trace]; and normal [white trace]). The complete lack of MSI for individuals with MCI and dementia is in keeping with our previous findings.^24^

Collectively, results reveal on average that: (1) those with normal cognitive status maintain the highest *magnitude of VSI* and APS scores that were negative for brain amyloid; (2) as cognitive impairment increases, the mean *magnitude of VSI* significantly decreased (*p* < 0.05; see also Figure 3A); and (3) individuals with dementia revealed the lowest *magnitude of VSI* and the highest APS.

## 4 DISCUSSION

In the current study, we provide strong support for the *magnitude of VSI* as a novel behavioral marker of AD‐associated amyloid pathology given its significant association with an established accurate and reliable biomarker of AD pathology: APS. Our linear regression, which controlled for many critical covariates associated with AD pathogenesis, revealed an independent significant inverse association between *magnitude of VSI* and APS. This key finding indicates that older adults at greater risk for AD‐associated amyloid pathology (i.e., those with higher APS scores) manifested significantly worse *magnitude of VSI*. In fact, on average, individuals with a greater likelihood of positive brain amyloid accumulation (i.e., those in the APS intermediate and APS positive groups) did not demonstrate any MSI whatsoever and were 94% more likely to be considered *non‐integrators* on the VSI test compared to individuals in the APS− group, who on the average manifested positive *magnitude of VSI* values (see Figure 2 and Table 1).

While the current experiment aimed to uncover associations of MSI with markers of AD‐associated amyloid pathology as support for VSI as a novel behavioral marker of AD pathology, we would be remiss to ignore its association with cognitive diagnoses. Findings from our sensitivity analysis revealed that individuals with normal cognitive function exhibited significantly larger *magnitude of VSI*, compared to individuals with MCI and dementia, who on average maintained zero or negative *magnitude of VSI* values indicative of poor MSI (see also Figure 3A). In fact, the percentage of *non‐integrators* (those with *magnitude of VSI* values ≤ 0) within each cognitive status group increased as cognitive impairment increased: with 35% in the normal group, 67% in the MCI group, and 73% in the dementia group. Collectively, these results are consistent with our previously published findings where evidence for robust, but differential VSI effects in aging was provided and further linked to several clinically meaningful outcomes.^19^, ^20^, ^21^, ^24^, ^50^ Specifically, our findings revealed that older adults with *magnitude of VSI* values > 0 (i.e., *integrators*) demonstrated better balance, faster gait velocity, and maintained lower incidence of falls, compared to *non‐integrators*. Further, we reported that older adults with MCI and dementia demonstrated significantly reduced *magnitude of VSI* compared to older adults without cognitive impairments, which was in turn associated with worse unipedal stance and spatial gait performance.^24^

We specifically chose a robust marker of amyloid pathology for AD that considers three critical contributors of AD risk factors including: age, Aβ42:40 concentration levels, and APOE4 proteotypes. However, to address concerns about whether the relationship between the *magnitude of VSI* (independent variable) and amyloid pathology could be due to chronological age, we ran a fully‐adjusted additional linear regression with the Aβ42:40 ratio as the dependent variable. Results revealed significant associations between *magnitude of VSI* and Aβ42:40 concentration levels (*β* = 0.13, *p* = 0.03), suggesting a significant association between VSI and amyloid pathology regardless of chronological age and other AD‐associated covariates.

Collectively, findings from the current study provide support for the *magnitude of VSI* as a novel, non‐invasive behavioral marker of AD‐associated amyloid pathology. While growing evidence suggests that Alzheimer's pathology, mainly Aβ, manifests in sensory association areas, more studies are needed to determine the precise location of these depositions and their integrated impact on cognitive, sensory, and motor functioning. While there is some evidence linking MSI to cognitive impairments using different types of multisensory combinations with varying levels of cognitive demands, previous investigations have not comprehensively examined the interplay of sensory, cognitive, and motor dysfunction in relation to AD progression using simple RT tests. Continued investigations are still needed to close these existing knowledge gaps, especially given Zhang et al.’s assertion that combined sensory impairment and AD progression could potentially perpetuate each condition.^32^

### 4.1 Limitations

These data were collected as part of a larger NIH study called the CCMA study, where a large proportion of the participants were White individuals and female and where representation of gender and ethnic groups was not equal but was representative of the larger Bronx catchment area. Also, the CCMA study did not aim to study AD as an outcome, and therefore data on the family history of AD was not collected. Continuous measures of somatosensory acuity (i.e., vibratory thresholds) were unfortunately not available for the entire study cohort, so our analyses could only control for self‐reported presence of neuropathy. Additionally, eye‐tracking during the MSI experiment was not available but given that the range of accuracy across groups and conditions was 88%–97%, it is unlikely that participants were not attending to the task at hand. We do however recognize these shortcomings and are currently striving to enroll a more culturally diverse and gender‐balanced cohort in our ongoing funded projects that investigate MSI using simple RT tests in normal and pathological aging, while also obtaining additional measures not available in the parent study.

### 4.2 Future directions

Poor balance is a major predictor of falls, a leading cause of injury and death in older Americans.^51^ Our research has revealed that better integration of visual‐somatosensory information, reflected as a larger magnitude of VSI, is associated with better balance and gait, as well as decreased risk of falls.^20^ However, our investigations to date do not fully determine the influence of early dementia stages on MSI processes and their contribution to mobility decline. Given previous and current findings, future studies are needed to determine the sensitivity of the VSI experiment in concurrently screening for risk of both falls and AD. As well, considerations should also be made for medication intake and how individual and combined usage could impact results.

The current study is in keeping with the recent NIA Alzheimer's Association (NIA‐AA) research framework,^52^ in so far as it looks at both cognitive status and Aβ probability scores which take into account Aβ42:40 concentration levels, chronological age, and APOE proteotypes. However, it would be helpful if future studies aim to distinguish AD symptomology (mere presence of mild cognitive impairment) and AD pathology (**A**β accumulation) while applying the **AT**(**N**) classification system [Aβ (**A**), tau (**T**), and neurodegeneration (**N**)] which will afford more direct assessments of neuropathologic changes. In the future, we also aim to establish whether the *magnitude of VSI* is an early biomarker of AD pathology, by examining associations of VSI with **A**β accumulation, plasma‐based total and phosphorylated **T**au, neurofilament (NfL), APOE, and multimodal neuroimaging measures of **N**eurodegeneration.

While the adverse effect of dementia and cognitive impairment on the relationship between multisensory functioning and motor outcomes has been highlighted here, the underlying functional and neuroanatomical neural substrates of VSI are not well‐established in healthy aging, let alone in neurodegenerative diseases like AD. In animals and young adults, efficient MSI depends on intact feedforward and feedback neural loops between cortical and subcortical regions.^53^ The PFC, a brain area instrumental in complex cognitive behaviors, also regulates the flexibility of MSI in young adults and primates.^6^, ^7^ Other key structures involved in MSI processes include the thalamus, which integrates sensory information through cortico‐cortical and cortical‐subcortical transmissions,^54^ and basal ganglia, which maintains connections with other subcortical regions involved in motor processes including brainstem and cerebellum, which play a role in cognition given PFC connections.^55^

Cortico‐cortical and cortico‐thalamic loops required for MSI are likely compromised in pathological aging. We believe that multisensory, motor and cognitive performance are linked to the integrity of these neural networks, given their reliance on prefrontal cortex. The PFC receives afferents from cortical and subcortical areas, including sensory, motor, association areas, and the thalamus, which are known to be implicated in multisensory processes.^56^ Recent studies provide support for a prominent role of dorsomedial and ventrolateral PFC in MSI processes ^6^, ^56^ and suggest that PFC serves as the “key driver of flexible multisensory behavior”; however, these studies do not examine VSI in older adults with MCI or preclinical AD and could prove valuable in determining whether PFC or other neural networks are integral for regulation of VSI in healthy and pathological aging.

The central hypothesis of our recently funded *VSI Study* is that preclinical AD is associated with neural disruptions in subcortical and cortical areas that concurrently modulate sensory, cognitive, and motor functions, resulting in mobility decline and incident falls in AD.^57^ We hypothesize that VSI likely requires activation of similar neural networks, especially those in the PFC, known to be compromised in dementia and MCI.^58^ A deeper understanding of the underlying neural correlates of VSI and their concurrent associations with cognitive and motor outcomes will foster development of novel multisensory interventions designed to target specific neural derailments, while significantly augmenting existing interventions to prevent disability, optimize independence, and further aim to enhance quality of life for older adults at‐risk for AD.

## CONFLICT OF INTEREST STATEMENT

J.R.M. has a financial interest in JET Worldwide Enterprises Inc., a digital health startup spun out of research conducted at the Albert Einstein College of Medicine. Author disclosures are available in the Supporting Information.

## CONSENT STATEMENT

All participants provided written informed consent to the experimental procedures prior to inclusion in the study, which were performed in accordance with the ethical standards of *The Declaration of Helsinki* and approved by the institutional review board at the Albert Einstein College of Medicine.

## Supporting information



Supporting Information
